# Supplementary material for: Intervention for Children with Obesity and Overweight and Motor Delays from Low-Income Families: Fostering Engagement, Motor Development, Self-Perceptions, and Playtime
Source: Int J Environ Res Public Health. 2022 Feb 22;19(5):2545. doi: 10.3390/ijerph19052545 (PMC8909693; doi:10.3390/ijerph19052545)
Supplement: Supplementary file 1 [file ijerph-19-02545-s001.zip › ijerph-1579378-supplementary.pdf]

Supplementary Table S1. Spare Time: Descriptive scores, independent and dependent t-tests & Delta comparisons for MCG and CG

**Children Spare Time**	**Children with Obesity/Overweight**				**Children with Adequate Weight**			
	M(SD)		Between groups		M(SD)		Between groups	
	MCG	CG	*p*	Cohen's D	MCG	CG	*p*	Cohen's D
**Daily time playing (minutes)**								
Pre	140.00(28.82)	138.95(28.65)	1.00	.04	141.82(30.27)	155.29(30.44)	1.00	.47
Post	177.77(20.25)	137.90(28.00)	< .0001*	1.67##	169.00(24.27.)	158.82(29.55)	1.00	.74
Within group *p*	< .0001*	1.00			.016*	.332		
Δ Scores	37.77(29.53)	1.05(20.00)	< .0001*	1.46##	27.18(31.34)	3.53(14.55)	.095	1.13#
**Daily time using computer (minutes)**								
Pre	97.77(85.99)	88.42(80.91)	---	---	85.45(72.16)	76.00(65.99)	---	---
Post	75.55(65.71)	85.26(78.27)	---	---	76.36(60.54)	72.00(60.85)	---	---
Within group *p*	.001*	.331			.598	.334		
Δ Scores	-22.22(29.52)	-3.16(13.77)	.006*	.91#	-9.09(55.40)	-4.00(15.49)	.772	.12
**Daily time watching TV (minutes)**								
Pre	168.89(23.75)	154.73(30.43)	.546	.54	158.18(30.27)	162.82(28.18)	1.00	.17
Post	133.33(25.42)	161.05(28.65)	.004*	1.06#	125.45(18.09)	162.35(28.00)	.003*	1.55#
Within group *p*	< .0001*	.163			.006*	1.00		
Δ Scores	-35.56(30.04)	6.32(18.92)	< .0001*	1.15#	-32.73(31.33)	.47(21.21)	.010*	1.31#

Note. * Significant result; # large effect size; ## very large effect size; --- statistics were not conducted since the interaction groups x time were non significant

Supplementary Table S2. Perceived Competence: Descriptive scores, independent and dependent t-tests & Delta comparisons for MCG and CG

PICTORIAL SCALE	**Children with Obesity/Overweight**				**Children with Adequate Weight**			
	M(SD)		Between groups		M(SD)		Between groups	
	MCG	CG	*p*	Cohen's D	MCG	CG	*p*	Cohen's D
**Perceived Social Acceptance**								
Pre	16.26(2.89)	17.74(4.09)	---	---	15.73(3.87)	16.24(3.11)	---	---
Post	18.85(2.61)	17.47(5.12)	---	---	19.45(2.30)	16.90(4.24)	---	---
Within group *p*	< .0001*	.813			.001*	.383		
Δ Scores	2.59(1.69)	-.27(4.77)	.021*	.72#	3.72(2.53)	.66(3.42)	.008*	1.00##
**Perceived Motor Competence**								
Pre	18.89(2.65)	19.84(2.24)	.195	.39	19.73(1.68)	20.43(2.50)	.354	.32
Post	21.63(2.00)	20.10(2.73)	.046*	.67#	22.27(1.68)	20.57(1.32)	.010*	1.21###
Within group *p*	< .0001*	.617			< .0001*	.719		
Δ Scores	2.74(1.35)	.26(2.26)	< .0001*	1.43###	2.54(1.21)	.14(1.80)	< .0001*	1.52###
**Global Self-Worth**								
Pre	54.89(5.59)	58.74(5.89)	.032*	.69#	55.09(4.85)	56.24(6.33)	.573	.20
Post	62.22(5.15)	59.17(7.37)	.127	.51	62.63(3.64)	58.00(5.88)	.010*	.91##
Within group *p*	< .0001*	.755			< .0001*	.201		
Δ Scores	7.33(2.73)	.43(5.79)	< .0001*	1.66###	7.54(3.24)	1.76(6.12)	.001*	1.12##

Note. * Significant result; # medium, ## large, and ### very large effect sizes; --- statistics were not conducted since the interaction groups x time were non significant

Supplementary Table S3. BMI, Waist circumference, and TGMD-2: Descriptive scores, independent and dependent t-tests & Delta comparisons for MCG and CG

BMI & TGMD-2	Children with Obesity/Overweight				Children with Adequate Weight			
	M(SD)		Between groups		M(SD)		Between groups	
	MCG	CG	*p*	Cohen's D	MCG	CG	*p*	Cohen's D
**BMI**								
Pre	21.88(3.79)	20.63(3.82)	1.00	.34	15.73(1.27)	15.57(.90)	1.00	.16
Post	20.45(3.52)	20.06(4.05)	1.00	.11	15.47(1.17)	15.82(.90)	1.00	.36
Within group *p*	< .0001*	.032*			.526	.214		
Δ Scores	-1.43(1.60)	-.57(1.09)	.039*	$.61^{\#}$	-.26(1.30)	.25(.87)	.267	.01
**Waist circumference**								
Pre	72.30(10.25)	70.29(11.16)	---	---	58.68(3.80)	59.90(4.43)	---	---
Post	70.42(9.53)	69.66(10.84)	---	---	57.41(2.56)	58.31(3.15)	---	---
Within group *p*	.049*	.271			.453	.271		
Δ Scores	-1.88(4.75)	-.63(3.58)	.313	.32	-1.27(3.62)	-1.59(3.67)	.815	.09
**Locomotor**								
Pre	21.62(5.18)	17.58(4.48)	.042*	.84	24.00(6.20)	17.85(3.94)	.007*	1.32
Post	34.59(4.22)	21.21(6.03)	< .0001*	$2.71^{\#\#\#}$	35.18(2.40)	21.38(4.62)	< .0001*	$3.55^{\#\#\#}$
Within group *p*	< .0001*	.019*			< .0001*	.003*		
Δ Scores	12.97(4.51)	3.63(6.15)	< .0001*	$1.82^{\#\#}$	11.18 (6.90)	3.53(4.86)	.005*	$1.44^{\#\#}$
**Object Control**								
Pre	18.85(5.28)	18.21(4.31)	.100	.13	22.73(7.19)	19.33(4.14)	.466	.66
Post	33.63(4.32)	21.31(4.16)	< .0001*	$2.96^{\#\#\#}$	32.73(4.98)	22.00(4.72)	< .0001*	$2.30^{\#\#\#}$
Within group *p*	< .0001*	.013*			< .0001*	.015*		
Δ Scores	14.78(5.66)	3.10(4.93)	< .0001*	$2.22^{\#\#\#\#}$	10.00(4.79)	2.67(4.57)	< .0001*	$1.59^{\#\#}$

Note. * Significant result; $^{\#}$ medium, $^{\#\#}$ very large, and $^{\#\#\#}$ huge effect sizes; --- statistics were not conducted since the interaction groups x time were non significant

Supplementary Table S4. Children Motor Engagement within the context: Descriptive scores, independent and dependent t-tests & Delta comparisons for MCG

**Motor Engagement**		**Mastery Climate in Context: M(SD) & Statics Results**			
		**Obesity/ Overweight**	**Adequate Weight**	**Between groups**	
				p	Cohen's D
Appropriate motor engagement: With Success	Pre	.30(.46)	.54(.82)	---	---
	Post	5.89(1.97)	6.09(1.45)	---	---
	Within group *p*	< .0001*	< .0001*		
	Δ Scores	5.59(1.97)	5.55(1.37)	.934	.03
Appropriate motor engagement: Without Success	Pre	5.44(2.19)	5.82(1.25)	---	---
	Post	5.04(1.87)	4.73(1.62)	---	---
	Within group *p*	.558	.119		
	Δ Scores	-.40(3.56)	-1.09(2.12)	.472	.22
Appropriate motor engagement: Moving around stations	Pre	.15(.53)	.0(0)	.161	.34
	Post	.04(.19)	.54(1.21)	.196	.78
	Within group *p*	.185	.167		
	Δ Scores	-.11(.42)	.54(1.21).	.108	.60
Non- appropriate motor engagement: Free Playing	Pre	.74(.94)	.73(1.10)	---	---
	Post	.22(.51)	.09(.30)	---	---
	Within group *p*	.017*	.046*		
	Δ Scores	-.52(1.05)	-.64(.92)	.736	.47
Non-appropriate motor engagement: Changing Tasks	Pre	1.70(1.03)	1.73(1.00)	---	---
	Post	.48(.75)	.60(.81)	---	---
	Within group *p*	< .0001*	.010*		
	Δ Scores	-1.22(1.39)	-1.13(1.14)	.766	.10
Non-engage in motor tasks: Organizing equipment & Instruction	Pre	1.30(1.14)	1.09(1.04)	---	---
	Post	.15(.36)	.18(.60)	---	---
	Within group *p*	< .0001*	.033*		
	Δ Scores	-1.15(1.29)	-.91(1.22)	.597	.19
Non- engage in motor tasks & inappropriate behavior: Distractions	Pre	2.37(1.52)	1.18(1.08)	.012*	.87#
	Post	.18(.39)	.09(.30)	.434	.25
	Within group *p*	< .0001*	.003*		
	Δ Scores	-2.19(1.47)	-1.09(.94)	.011*	.83#
Non-engage in motor tasks & inappropriate behavior: Conflicts	Pre	.22(.70)	.77(.90)	---	---
	Post	0(0)	.09(.30)	---	---
	Within group *p*	.110	.026*		
	Δ Scores	-.22(.70)	-.68(.81)	.156	.59

Note. * Significant result; # large effect size; --- statistics were not conducted since the interaction groups x time were non significant.
